# Sequential ejaculated- and testicular-sperm ICSI cycles in couples with prior ICSI failure: a within-pair analysis of embryo quality, euploidy, and live birth

**DOI:** 10.3389/fmed.2026.1903479

**Published:** 2026-10-28

**Authors:** Ayşen Yücetürk, Nuri Peker, Esra Tığlı, Tansu Güdelci, Özge Karaosmanoğlu, Burak Elmas, İlke Özer Aslan, Bülent Tıraş

**Affiliations:** 1 Department of Obstetrics and Gynecology, Assisted Reproductive Technologies Unit, Acibadem Maslak Hospital, Istanbul, Türkiye; 2 Department of Obstetrics and Gynecology, Kütahya Health Sciences University, Kütahya, Türkiye; 3 Department of Urology, Assisted Reproductive Technologies Unit, Acibadem Maslak Hospital, Istanbul, Türkiye; 4 Department of Obstetrics and Gynecology, Assisted Reproductive Technologies Unit, Acibadem Mehmet Ali Aydınlar University, Istanbul, Türkiye

**Keywords:** blastocyst quality, intracytoplasmic sperm injection, live birth, paired cohort study, preimplantation genetic testing for aneuploidy, recurrent ICSI failure, testicular sperm aspiration

## Abstract

**Introduction:**

Persistent poor outcomes after intracytoplasmic sperm injection (ICSI) occur in a clinically significant subset of couples despite reassuring conventional semen analysis. In such couples, testicular sperm aspiration (TESA) may be undertaken in a subsequent cycle on clinical and embryological grounds. We assessed whether TESA-ICSI improves embryo-quality indicators and live birth rates compared with EJAC-ICSI in couples whose initial EJAC-ICSI cycle did not result in a live birth.

**Methods:**

This retrospective paired cohort study included 58 sequential ICSI cycle pairs at a single tertiary IVF center. Each pair comprised an initial ICSI cycle with ejaculated sperm (EJAC-ICSI) that had not resulted in a live birth, followed by a subsequent TESA-ICSI cycle in the same couple; the cycle pair was the unit of analysis. The primary outcome was live birth rate per cycle; secondary outcomes included fertilization, blastulation, Grade A blastocyst formation, PGT-A, clinical pregnancy, miscarriage, and live birth per embryo transfer. Paired comparisons used McNemar exact and Wilcoxon signed-rank tests.

**Results:**

Total oocyte, mature oocyte, 2PN, and blastocyst counts were similar between paired cycles (all *p* > 0.05), while the fertilization rate was lower in TESA-ICSI cycles at the oocyte level (71.3% vs. 79.7%; *p* = 0.001). By contrast, the blastulation rate per 2PN was higher in TESA-ICSI cycles (*p* = 0.008), as was the Grade A blastocyst rate both at the per-embryo level (EJAC: 2.4% vs. TESA: 8.9%; *p* = 0.0006) and at the per-patient level (*p* = 0.0032). PGT-A euploidy increased from 27.9% to 65.0% (*p* = 0.0009), the clinical pregnancy rate from 6.9% to 31.0% (*p* = 0.0026), and the live birth rate from 0% to 29.3% (absolute risk difference 29.3%; number needed to treat 3.4) (*p* = 1.5 × 10^−5^), while the miscarriage rate per clinical pregnancy fell from 4/4 to 1/18 (*p* = 0.0007).

**Conclusions:**

In couples whose prior EJAC-ICSI cycle had not resulted in a live birth, a subsequent TESA-ICSI cycle in the same couple was associated with significant improvements in embryo quality, euploidy, and live birth without change in embryo numbers. These findings support TESA-ICSI as a reasonable therapeutic option in this clinical scenario.

## 1 Introduction

Intracytoplasmic sperm injection (ICSI) has become an integral component of assisted reproductive technology (ART) globally, with the total number of infants born by ICSI exceeding 8 million by 2024 (1, 2). Male factor infertility, present in approximately half of all infertile couples (3, 4), can today be largely addressed through ICSI, which bypasses many barriers to fertilization. Despite these advances, a clinically significant subset of couples experience persistent poor outcomes after ICSI, including impaired embryo development, failed implantation, recurrent pregnancy loss, and ultimately failure to achieve a live birth, even when conventional semen analysis appears reassuring (5, 6). These cases highlight a fundamental limitation of standard semen evaluation: it does not capture the molecular integrity of the sperm genome. Sperm DNA fragmentation (SDF) is the most extensively studied of these molecular characteristics and has been associated with post-fertilization embryonic competence (3, 7). SDF testing is nevertheless not part of routine practice in many centers, and management decisions in couples with repeated ICSI failure are therefore usually made on clinical and embryological grounds (8).

Testicular spermatozoa, retrieved prior to epididymal transit, have been reported to carry substantially less DNA fragmentation than ejaculated sperm. Building on early clinical observations by Greco and colleagues nearly two decades ago (9), the use of testicular sperm for ICSI in non-azoospermic men with recurrent failure is now supported by recent meta-analyses (10), which report approximately 25% lower SDF and higher live birth rates with TESA-ICSI compared with continued use of ejaculated sperm (11, 12). Recurrent ICSI failure is recognized as an independent indication for TESA-ICSI, regardless of whether formal SDF testing has been performed (8). The evidence, however, is not uniform. A recent exploratory randomized trial by Paladino and colleagues (8) showed no significant live birth benefit, and a Turkish series comparing microdissection testicular sperm extraction (m-TESE) with ejaculated sperm reported superior outcomes with ejaculated sperm in selected recurrent-failure cases (13). Yet, most current comparisons are between subjects and exhibit intrinsic selection bias. As far as we know, there are still limited within-subject (paired) designs that incorporate embryo development, pre-implantation genetic testing for aneuploidy (PGT-A), and live birth within a single dataset.

To address these limitations, we conducted a retrospective paired cohort study of 58 sequential ICSI cycle pairs. Each pair comprised an initial ICSI cycle performed with ejaculated sperm (EJAC-ICSI) that did not result in a live birth, due either to failure to obtain transferable-quality embryos or to unsuccessful embryo transfer, followed by a subsequent ICSI cycle performed with sperm retrieved by TESA (TESA-ICSI) in the same couple. The cycle pair was used as the unit of analysis, with each pair providing its own internal control, thereby eliminating between-subject heterogeneity in female and clinical factors. The primary outcome was the live birth rate per cycle; secondary outcomes included fertilization, blastulation, Grade A blastocyst formation, PGT-A results, clinical pregnancy, miscarriage, and live birth per embryo transfer. We hypothesized that, in this clinically refractory population, TESA-ICSI would improve embryo-quality indicators and live birth rates compared with EJAC-ICSI.

## 2 Methods

### 2.1 Study design

This retrospective, cohort study was conducted at the IVF Centre of Acıbadem Maslak Hospital, Istanbul, Türkiye. Clinical and embryological data were retrieved from the center's electronic IVF laboratory and clinical records databases. Cycle pairs were accrued consecutively between July 2017 and April 2024; this window reflects the accrual period across all 58 couples and does not represent the interval between the paired EJAC-ICSI and TESA-ICSI cycles within any individual couple, which is separately reported in Section 2.3.

### 2.2 Ethical approval and data confidentiality

The study protocol was approved by the Medical Research Evaluation Board (ATADEK) of Acıbadem Mehmet Ali Aydınlar University (approval no. 2026-07/280; 9 April 2026) and was conducted in accordance with the principles of the Declaration of Helsinki. The ethics committee waived the requirement for informed consent due to the retrospective nature of the study. All data were de-identified and analyzed using anonymous identification codes; patient names, medical record numbers, and any other identifying information were excluded from analysis and reporting.

### 2.3 Patient selection

The study population consisted of couples who had been referred to our center after at least one unsuccessful IVF/ICSI attempt elsewhere, and in whom an ICSI cycle using ejaculated sperm (EJAC-ICSI) had been performed first and had not resulted in a live birth and who subsequently underwent a consecutive ICSI cycle using sperm obtained via testicular sperm aspiration (TESA-ICSI).

Inclusion criteria:

- Prior failed EJAC-ICSI cycle, defined as either failure to obtain a transferable embryo, no implantation following embryo transfer, or pregnancy loss prior to live birth.
- Subsequent consecutive TESA-ICSI cycle in the same couple.

Exclusion criteria:

- Females aged under 18 and over 45 years at the time of the EJAC-ICSI cycle.
- Azoospermia, uterine anomalies, prior uterine surgery, intrauterine synechiae, severe oligospermia, and cryptozoospermia.

A total of 58 cycle pairs were included in the final analysis.

Within each couple, the TESA-ICSI cycle was initiated after the failed EJAC-ICSI cycle, following luteal recovery and appropriate endometrial preparation, in accordance with routine clinical practice. Both cycles for any given couple were performed within the same institutional laboratory environment. The NGS platform used for PGT-A did not change during the study period.

### 2.4 Ovarian stimulation protocol

Controlled ovarian stimulation was performed using a flexible gonadotropin-releasing hormone (GnRH) antagonist protocol in all cycles. On the second or third day of menstruation, antral follicle count (AFC) was assessed by transvaginal ultrasonography. Stimulation was initiated on the same day with a combination of recombinant follicle-stimulating hormone (Gonal-F®; Merck Serono, Switzerland) and luteinizing hormone activity (Meriofert®; IBSA, Switzerland); daily doses were individualized according to patient age, body mass index, and AFC. The first follicular monitoring was performed five days after initiation; thereafter, monitoring was performed every 1–2 days according to follicular response. A GnRH antagonist (cetrorelix acetate, Cetrotide®; Merck Serono) was added in a flexible regimen when the leading follicle reached ≥14 mm in diameter or when serum estradiol (E₂) exceeded 300–500 pg/mL. Final oocyte maturation was triggered with a dual trigger consisting of human chorionic gonadotropin (hCG) and triptorelin acetate when at least three follicles reached ≥17 mm in mean diameter. However, in patients diagnosed with polycystic ovary syndrome (PCOS), triggering was performed using only triptorelin acetate. Oocyte pick-up (OPU) was performed 36 h after trigger administration under intravenous sedation.

### 2.5 Sperm procurement

#### 2.5.1 Ejaculated sperm

For the EJAC-ICSI cycle, semen samples were obtained on the day of oocyte retrieval by masturbation following 2–5 days of sexual abstinence. Samples were analyzed according to the World Health Organization (WHO) 2021 (sixth edition) laboratory manual for the examination and processing of human semen, with reference values for volume (∼1.4 mL), sperm concentration (∼16 × 10^6^ mL), total sperm number (∼39 × 10^6^ per ejaculate), progressive motility (∼30%), and normal morphology (∼4%).

#### 2.5.2 Testicular sperm aspiration (TESA)

For the TESA-ICSI cycle, testicular sperm were obtained on the day of oocyte retrieval by percutaneous testicular sperm aspiration. Depending on patient preference and level of procedural anxiety, the aspiration was performed either under local anesthesia or under short-acting general anesthesia; the latter option was offered to minimize the risk of sudden patient movement during the procedure, which could cause hematoma or damage to intratesticular structures. One to three punctures were performed per procedure using a 21-gauge butterfly needle; the median total procedural time was 15 min, and the median duration from patient sedation to full recovery was approximately 40 min. No procedural complications, including hematoma, infection, or the need for reintervention, were observed in any of the 58 TESA procedures.

The aspirate was transferred to a sterile dish containing sperm wash medium and mechanically minced with an insulin needle to release spermatozoa entrapped within tissue fragments, without enzymatic digestion. Motile spermatozoa were selected for immediate use in ICSI whenever available; for cycles in which only immotile spermatozoa were retrieved, sperm motility was induced using SpermMobil (Gynemed, Lensahn, Germany) prior to ICSI.

Beyond the prior unsuccessful cycle required for inclusion, the clinical indication for switching to TESA-ICSI was an aneuploid PGT-A result in the initial EJAC-ICSI cycle (*n* = 30), persistent poor embryo development (*n* = 20), or more than one of these (*n* = 8).

#### 2.5.3 Intracytoplasmic sperm injection (ICSI) and embryo culture

Oocytes were retrieved at OPU and incubated for 2–3 h prior to enzymatic denudation. ICSI was performed exclusively on oocytes at the metaphase II (MII) stage of meiosis. Fertilization assessment was performed 16–18 h after ICSI; oocytes displaying two clearly visible pronuclei (2PN) were classified as normally fertilized.

Embryos were cultured in single-step medium (NX Complete Single Culture Medium; Fujifilm Irvine Scientific, USA) in conventional benchtop incubators under reduced (5%) O₂ tension at 37 °C until the blastocyst stage.

### 2.6 Embryo assessment and grading

Blastocyst quality was assessed on day 5 (D5) and, when relevant, on day 6 (D6) post-fertilization, according to the system described by Gardner and Schoolcraft. Each blastocyst was assigned a composite score consisting of (i) an expansion grade from 1 to 6, (ii) an inner cell mass (ICM) grade (A, B, or C), and (iii) a trophectoderm (TE) grade (A, B, or C) (14). For the present analysis, blastocysts were classified into three composite quality categories:

Grade A: Expansion grade ≥ 3 with an ICM grade of A and a TE grade of A or B, or vice versa.
Grade B: Expansion grade ≥ 3 with ICM and TE grades of BB, BC, or CB.
Grade C: Blastocysts with both ICM and TE graded C (i.e., CC), or with an expansion grade < 3.

### 2.7 Preimplantation genetic testing for aneuploidy (PGT-A)

Preimplantation genetic testing for aneuploidy (PGT-A) was offered to couples with any of the following prespecified indications: advanced maternal age (≥38 years), two or more prior failed ICSI cycles, a history of poor embryo development, or recurrent pregnancy loss. PGT-A was performed in at least one cycle in 50 of the 58 couples (86.2%): in the EJAC-ICSI cycle in 43 couples and in the TESA-ICSI cycle in 40. It was performed in both cycles in 33 couples, in the EJAC-ICSI cycle only in 10, and in the TESA-ICSI cycle only in 7; in 8 couples PGT-A was not performed in either cycle. The recorded indications were two or more prior failed ICSI cycles (*n* = 13), patient request (*n* = 12), advanced maternal age (*n* = 4), recurrent pregnancy loss (*n* = 2), a history of poor embryo development (*n* = 1), and a combination of two or more of these indications (*n* = 19); no indication was recorded for 7 couples. In cycles in which PGT-A was not performed, no blastocyst suitable for trophectoderm biopsy was available, or a day-3 transfer was undertaken. Trophectoderm biopsies were obtained from D5 or D6 expanded/hatching blastocysts and analyzed by next-generation sequencing (NGS) for 24-chromosome aneuploidy screening. The NGS platform did not change during the study period. Biopsied blastocysts were vitrified pending genetic results, which were classified as euploid or aneuploid. The indications for PGT-A did not change during the study period, although the overall number of cycles in which PGT-A was performed increased over time, consistent with international trends in PGT-A utilization.

### 2.8 Embryo transfer policy

All embryo transfers were performed in hormone replacement therapy (HRT) cycles as frozen-thawed transfers. Blastocysts were transferred whenever available; in five cycles (four EJAC-ICSI and one TESA-ICSI) no blastocyst developed and a day-3 cleavage-stage transfer was performed instead. Embryo selection followed a hierarchical algorithm: when PGT-A had been performed, euploid embryos were transferred first; thereafter, embryos were selected sequentially according to Gardner grade, with the highest-quality embryo transferred first.

The number of embryos transferred was determined in accordance with Turkish Ministry of Health regulations: in patients <35 years of age, single embryo transfer (SET) was performed in the first and second attempts, with the option of double embryo transfer (DET) permitted from the third attempt onwards; in patients ≥35 years, DET was permitted. The transfer of three or more embryos is prohibited under national legislation and was not performed.

### 2.9 Endometrial preparation and luteal support

Frozen embryo transfer (FET) cycles were prepared using an HRT protocol with pituitary down-regulation. A GnRH agonist (leuprolide acetate, Lucrin Depot® 3.75 mg) was administered on day 21 of the cycle preceding HRT. Following the onset of menstruation, baseline transvaginal ultrasonography and serum E_2_ and progesterone (P_4_) measurements were performed. If hormone values were within target ranges, oral estradiol hemihydrate (Estrofem®, 2 mg tablets) was commenced in escalating dosing: 2 mg twice daily for 5 days, 2 mg three times daily for the next 4 days, and 4 mg twice daily for the subsequent 5 days. Fifteen days after the initiation of estradiol treatment, endometrial thickness was reassessed and E₂ and P₄ levels were repeated. When endometrial thickness was ≥7 mm with P_4_ < 1.5 ng/mL and E_2_ > 150 pg/mL, luteal support was initiated with subcutaneous progesterone (Progestan Dex® 2 ampoules once daily) combined with vaginal micronized progesterone capsules (Progestan® 200 mg, 400 mg three times daily). Blastocyst transfer was performed on the sixth day following the initiation of progesterone supplementation.

### 2.10 Outcome definitions

Fertilization rate was calculated as the number of 2PN zygotes divided by the number of MII oocytes inseminated.

Blastulation rate (D5/2PN) was calculated as the number of day-5 blastocysts divided by the number of 2PN zygotes.

Clinical pregnancy was defined as ultrasonographic visualization of one or more of the following: a gestational sac, yolk sac, or fetal pole (with or without cardiac activity). Biochemical pregnancy, defined as a positive serum *β*-hCG without subsequent ultrasonographic confirmation, was not included in the clinical-pregnancy definition.

Miscarriage was defined as a clinically confirmed pregnancy loss prior to 20 weeks of gestation.

Live birth (the primary outcome) was defined as the delivery of at least one liveborn infant of ≥24 completed weeks of gestation.

### 2.11 Statistical analysis

Statistical analyses were performed using GraphPad Prism version 9.0.1 (GraphPad Software, San Diego, CA, USA) and jamovi version 2.3 with the cycle pair as the unit of analysis (*n* = 58). After conducting normality testing using the Shapiro–Wilk method, continuous variables were summarized as medians (IQR) with ranges and compared between paired cycles using the Wilcoxon matched-pairs signed-rank test, which provided exact two-sided *p*-values. Unpaired comparisons restricted to cycles in which embryo transfer was performed were analyzed with the Mann–Whitney U test. Paired binary outcomes (embryo transfer, clinical pregnancy, and cycle-level live birth) were analyzed with the McNemar exact test; outcomes whose denominators differed between cycles (per-transfer live birth and per-clinical-pregnancy miscarriage) were analyzed with Fisher's exact test. Blastocyst quality (Grade A vs. combined Grade B and C blastocysts) was assessed using two complementary approaches: (i) a per-embryo Fisher's exact test, treating each blastocyst as an independent observation across the two cycle types, and (ii) a patient-level Wilcoxon matched-pairs signed-rank test on within-cycle Grade A proportions, the latter accounting for within-subject clustering.

Effect sizes are reported with 95% confidence intervals computed using the Newcombe/Wilson method (proportion differences), the Koopman asymptotic-score method (relative risk), the Baptista-Pike method (odds ratios), and the Wilson/Brown method (individual proportions); number-needed-to-treat (NNT) was derived as the reciprocal of the risk difference. For paired binary comparisons, the conditional odds ratio (b/c) is reported. Live birth rate per cycle was the primary outcome; all other analyses were considered secondary or exploratory, and no formal multiple-comparison adjustment was applied. A two-sided *p* < 0.05 was considered statistically significant. Sensitivity analyses were performed using the McNemar exact test, restricted to couples whose paired cycles were separated by ≤6 months (*n* = 35) and ≤12 months (*n* = 50).

## 3 Results

The 58 cycle pairs included in the study consisted of cases with a median (IQR) female age of 33.0 (28.0–38.25) years and an age range of 22–44 years. All cases first underwent an ICSI cycle using ejaculated sperm (EJAC-ICSI), followed sequentially by an ICSI cycle using sperm obtained via testicular sperm aspiration (TESA-ICSI).

### 3.1 Ovarian response, fertilization, and embryo development

No statistically significant differences were observed between the two cycles in terms of total oocyte count, metaphase II (MII) oocyte count, and fertilized oocyte (2PN) count (all *p* > 0.05; Table 1). The fertilization rate (2PN/MII) was 0.82 (0.70–1.00) in the EJAC-ICSI cycle and 0.75 (0.56–0.94) in the TESA-ICSI cycle; this difference was not significant in the paired patient-level analysis (Wilcoxon paired, exact *p* = 0.081). When all injected MII oocytes were pooled, however, the fertilization rate was lower in TESA-ICSI cycles (418/586 [71.3%] vs. 467/586 [79.7%]; Fisher's exact *p* = 0.001).

**Table 1:** Paired comparison of inter-cycle interval, ovarian response, fertilization, and embryo development parameters between EJAC-ICSI and TESA-ICSI cycles (*n* = 58).

Variable	EJAC-ICSI Median (IQR)	TESA-ICSI Median (IQR)	Min-Max (EJAC/TESA)	p
Interval between paired cycles (months)	6.0 (3.0–9.0)	-	[2–24]	-
Total oocytes	15.0 (10.0–19.0)	14.0 (7.75–22.0)	[1–33]/[3–37]	0.5143
MII oocytes	9.5 (6.75–13.25)	11.0 (5.0–14.0)	[1–26]/[1–23]	0.9559
2PN	7.5 (4.0–11.25)	6.0 (3.0–10.25)	[1–22]/[1–20]	0.1754
2PN/MII (fertilization rate)	0.8214 (0.6961–1.0)	0.7500 (0.5649–0.9423)	[0.20–1.00]/[0.25–1.00]	0.0810
D5 total blastocysts	3.0 (0.0–7.0)	4.0 (2.0–7.0)	[0–14]/[0–14]	0.3544
D5/2PN (blastulation rate)	0.5 (0.0–0.738)	0.646 (0.466–0.778)	[0.000–1.000]/[0.000–1.000]	**0** **.** **008**
D6 total blastocysts	0.0 (0.0–1.0)	0.0 (0.0–0.25)	[0–13]/[0–7]	0.059

Continuous variables are presented as median (interquartile range) with minimum–maximum values and were compared using the Wilcoxon matched-pairs signed-rank test with exact two-sided *p* values. The interval between paired cycles is a single within-couple measure and is therefore reported once, with no between-group comparison. Significant *p* values are shown in bold.

In contrast, the blastocyst formation rate (D5/2PN) was significantly higher in the TESA-ICSI cycle [0.646 (0.466–0.778) vs. 0.5 (0.00–0.738); Wilcoxon paired, exact *p* = 0.008].

The total number of blastocysts on D5 was similar in both cycles (Figure 1); no statistically significant difference was observed between the two cycles regarding the total number of blastocysts on D6 (Figure 2).

**Figure 1:**
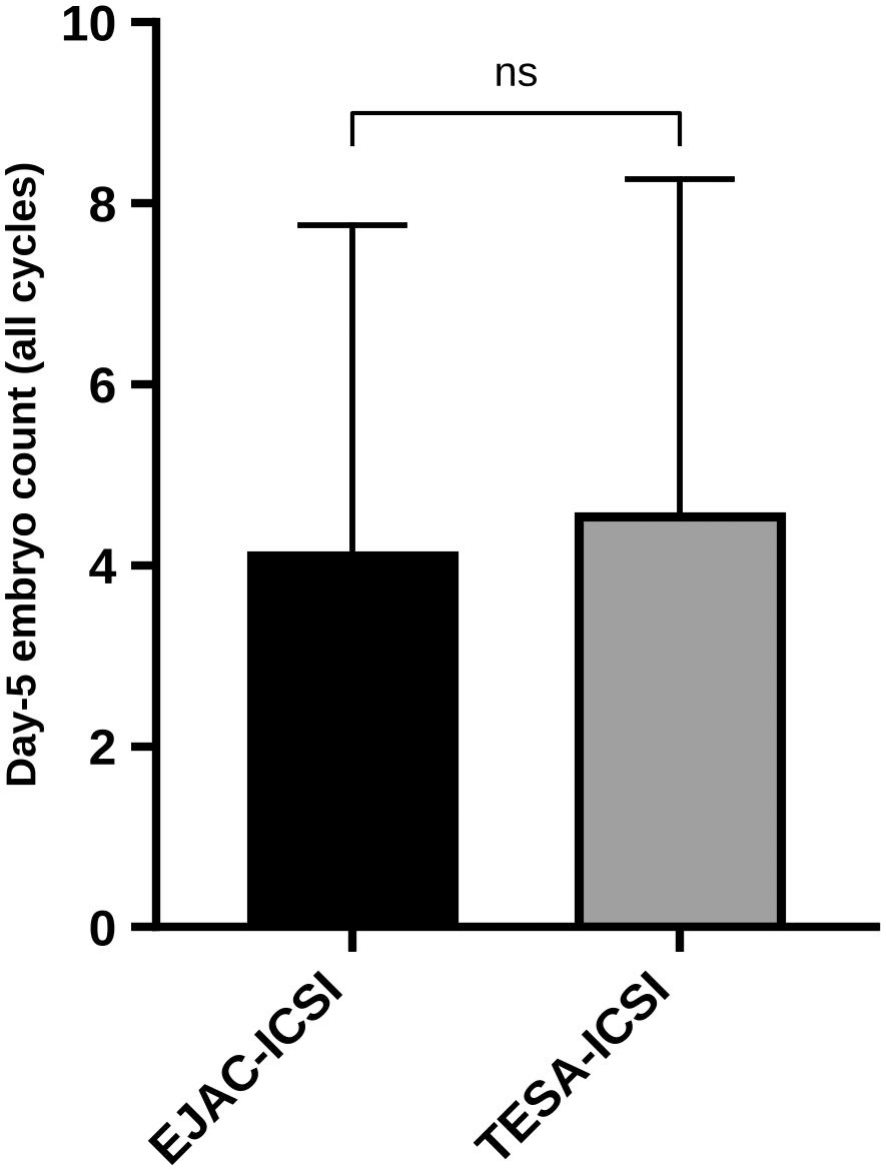
Comparison of the total number of D5 blastocysts between EJAC-ICSI and TESA-ICSI cycles in an analysis encompassing all cycles (*n* = 58 cycle pairs, mean ± SD). The Wilcoxon signed-rank test for matched pairs found no statistically significant difference (exact two-sided *p* = 0.354; ns).

**Figure 2:**
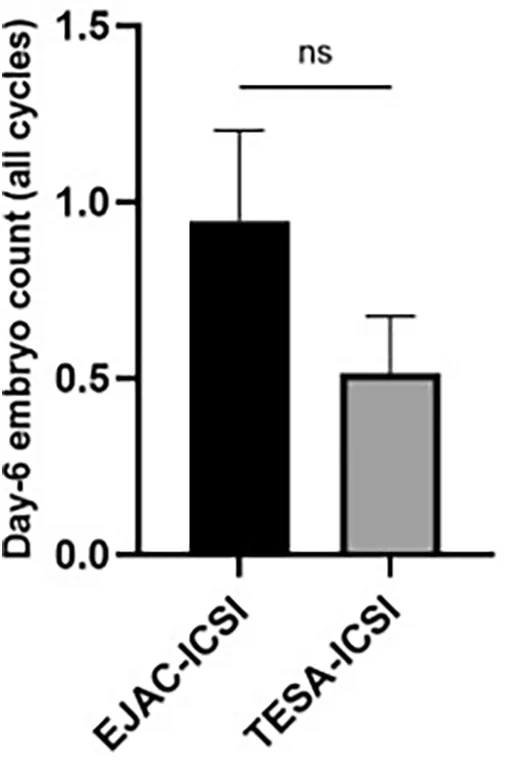
Comparison of the total number of D6 blastocysts between EJAC-ICSI and TESA-ICSI cycles in an analysis encompassing all cycles (*n* = 58 cycle pairs, mean ± SD). The Wilcoxon signed-rank test for matched pairs found no statistically significant difference (exact two-sided *p* = 0.059; ns).

### 3.2 Blastocyst quality

The distribution of blastocyst quality is presented in Table 2. While Grade B blastocysts were the most common category in both cycle types, the proportion of top-quality (Grade A) blastocysts was notably higher in the TESA-ICSI group.

**Table 2:** Distribution of blastocyst quality.

Day	Group	Grade A (%)	Grade B (%)	Grade C (%)	Total
D5	EJAC-ICSI	6 (2.5)	142 (59.7)	90 (37.8)	238
D5	TESA-ICSI	25 (9.5)	133 (50.8)	104 (39.7)	262
D6	EJAC-ICSI	1 (1.8)	31 (56.4)	23 (41.8)	55
D6	TESA-ICSI	1 (3.3)	24 (80.0)	5 (16.7)	30

Two supplementary analyses were conducted for the Grade A rate:

(i) Per-embryo analysis: When all blastocysts were combined across cases and treated as independent observations, the Grade A rate was 7/293 (2.4%) in the EJAC-ICSI cycle and 26/292 (8.9%) in the TESA-ICSI cycle; the between-group difference was statistically significant by Fisher's exact test (two-sided *p* = 0.0006; RR in favor of TESA=3.73 [95% CI: 1.69–8.30]; OR=3.99 [95% CI: 1.70–9.77]) (Figure 3).
(ii) Per-patient (matched) analysis to properly model the within-subject correlation of the design: The Grade A rate was calculated separately for each case (Grade A/total blastocysts), and a matched test was applied. In the 41 cases where ≥1 blastocyst was obtained in both cycles, the Grade A rate was significantly higher in the TESA-ICSI cycle (mean 11.3% ± 24.0 vs. 2.9% ± 7.3; sum of positive ranks 85, sum of negative ranks 6; Wilcoxon signed-rank test for matched pairs, exact two-sided *p* = 0.0032). The matching efficiency was significant (Spearman *ρ*=0.52, *p* = 0.0002) (Figure 4).

**Figure 3:**
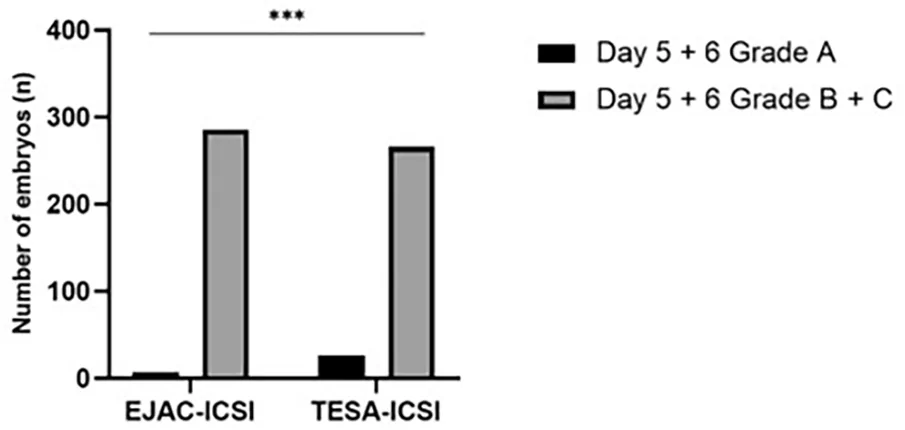
Comparison of grade A and grade B + C blastocysts (D5 and D6 combined) in EJAC-ICSI and TESA-ICSI cycles (absolute embryo counts). The Grade A rate is significantly higher in the TESA-ICSI cycle (7/293 [2.4%] vs. 26/292 [8.9%]; Fisher's exact test, two-sided *p* = 0.0006, *** indicates *p* < 0.001).

**Figure 4:**
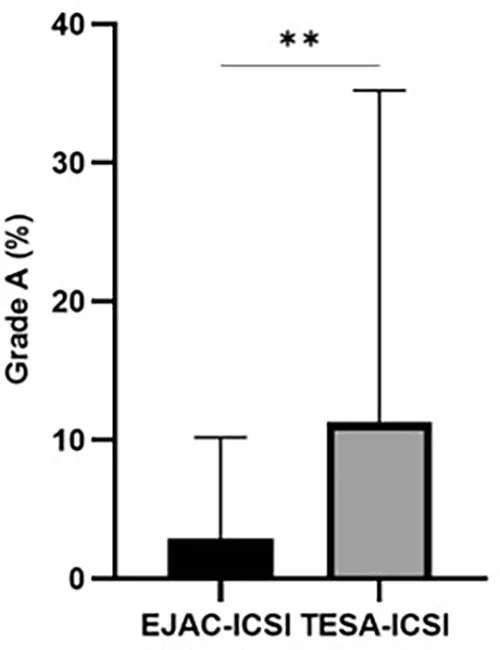
Paired patient-based comparison of the grade A blastocyst rate (%) (*n* = 41 cycle pairs, mean ± SD). The Grade A rate is significantly higher in the TESA-ICSI cycle (Wilcoxon signed-rank test for matched pairs, exact two-sided *p* = 0.0032; ** indicates *p* < 0.01).

The distribution across the three quality categories also differed significantly between cycle types (Grade A/B/C: 7/173/113 vs. 26/157/109; *χ*^2^, *p* = 0.003).

Similar results were obtained in the sensitivity analysis specific to D5: the matched D5 grade A rate was significantly higher in the TESA-ICSI cycle in 37 cases (mean 12.4% ± 24.9 vs. 3.3% ± 8.5; Wilcoxon matched-pairs signed-rank test, exact two-sided *p* = 0.0034). Since the number of D6 Grade A blastocysts was low in both groups (EJAC 1/55, TESA 1/30), no paired statistical comparison was performed; the data were reported descriptively only.

### 3.3 PGT-A results

Among cycles in which PGT-A was performed, aneuploidy was identified in 31 of 43 EJAC-ICSI cycles (72.1%) and in 14 of 40 TESA-ICSI cycles (35.0%); the corresponding euploidy rates were 12/43 (27.9%) and 26/40 (65.0%). The difference was statistically significant (Fisher's exact test, two-sided *p* = 0.0009; OR = 4.80) (Figure 5). Cycles in which PGT-A was not performed were excluded from this comparison, since no chromosomal result was available for them.

**Figure 5:**
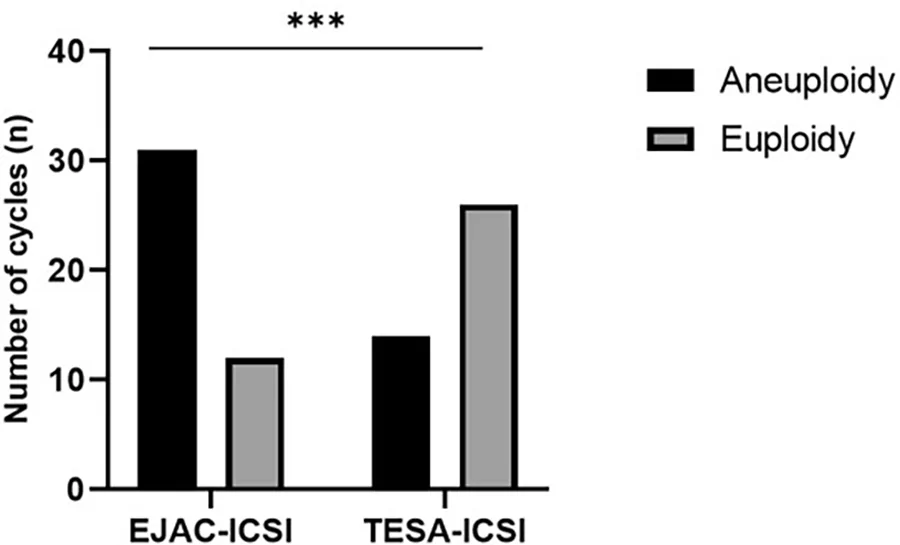
Comparison of PGT-A results (aneuploidy vs. euploidy) between EJAC-ICSI and TESA-ICSI cycles, restricted to cycles in which PGT-A was performed (absolute numbers of cycles). The aneuploidy rate was significantly lower in the TESA-ICSI cycle (14/40 [35.0%] vs. 31/43 [72.1%]; Fisher's exact test, two-sided *p* = 0.0009, *** indicates *p* < 0.001).

### 3.4 Clinical outcomes

#### 3.4.1 Embryo transfer

Embryo transfer (ET) was performed in 21 of 58 EJAC-ICSI cycles (36.2%) and in 30 of 58 TESA-ICSI cycles (51.7%). In the paired analysis, transfer was possible only in the TESA cycle in 12 cases, only in the EJAC cycle in 3 cases, and in both cycles in 18 cases. Because each cycle pair comprises two cycles from the same couple, the two arms are not independent patient groups. The ET rate was significantly higher in TESA-ICSI cycles (McNemar exact test, two-sided *p* = 0.035).

Characteristics of the transfer cycles are summarized in Table 3. Female age at transfer, the number of embryos transferred per transfer, and the proportion of double embryo transfers were similar between cycle types. Cleavage-stage transfer was required in 4 EJAC-ICSI cycles and 1 TESA-ICSI cycle because no blastocyst developed. Among transferred blastocysts, the proportion of Grade A embryos was higher in EJAC-ICSI cycles (14/26 [53.8%] vs. 10/38 [26.3%]; Fisher's exact *p* = 0.036), whereas a euploid embryo was transferred more often in TESA-ICSI cycles (26/30 [86.7%] vs. 12/21 [57.1%]; *p* = 0.024).

**Table 3:** Embryo transfer characteristics.

Characteristic	EJAC-ICSI	TESA-ICSI	p value
Cycles with embryo transfer	21/58 (36.2%)	30/58 (51.7%)	0.035
Female age at transfer, median (IQR)	30 (28–33)	31 (28–34)	1.00
Transferred embryos			
Total embryos transferred	32	42	-
Embryos per transfer, mean	1.52	1.40	0.39
Single/double embryo transfer	10/11	18/12	0.41
Developmental stage at transfer (cycles)			
Blastocyst	17 (81.0%)	29 (96.7%)	-
Cleavage stage (day 3)	4 (19.0%)	1 (3.3%)	-
Grade of transferred blastocysts (embryos)			
Grade A	14/26 (53.8%)	10/38 (26.3%)	0.036
Grade B	12/26 (46.2%)	22/38 (57.9%)	-
Grade C	0	6/38 (15.8%)	-
PGT-A status of the transferred embryo (cycles)			
Euploid	12/21 (57.1%)	26/30 (86.7%)	0.024
Aneuploid	5/21 (23.8%)	3/30 (10.0%)	-
Not tested (day-3 transfer)	4/21 (19.0%)	1/30 (3.3%)	-

McNemar exact test for transfer rate; Mann–Whitney U for age and embryos per transfer; Fisher's exact test for proportions. Cleavage-stage transfers are not graded by the Gardner system and are excluded from the blastocyst grade rows.

#### 3.4.2 Clinical pregnancy and miscarriage

The clinical pregnancy rate was significantly higher in the TESA-ICSI cycle compared to the EJAC-ICSI cycle [18/58 (31.0%) vs. 4/58 (6.9%); McNemar exact test, two-sided *p* = 0.0026; conditional OR = 5.67, 95% CI: 1.64–30.18]. In the paired analysis, clinical pregnancy was achieved only in the TESA cycle in 17 cases, only in the EJAC cycle in 3 cases, and in both cycles in 1 case (total EJAC pregnancies=3 + 1 = 4; total TESA pregnancies=17 + 1 = 18).

Among the 4 EJAC-ICSI clinical pregnancies, all 4 ended in miscarriage (4/4, 100%), whereas only 1 of the 18 TESA-ICSI clinical pregnancies resulted in miscarriage (1/18, 5.6%; Fisher's exact test, two-sided *p* = 0.0007). Because no event-free outcomes occurred in the EJAC arm, the odds ratio was not estimable; the Baptista-Pike lower 95% confidence limit was 6.59.

#### 3.4.3 Live birth

Live birth, the primary endpoint of the study, was not achieved in any of the cases in the EJAC-ICSI cycle [0/58 (0.0%; 95% Wilson CI: 0.0%–6.2%)]; it was observed in 17 cases in the TESA-ICSI cycle [17/58 (29.3%; 95% Wilson CI: 19.2%–42.0%)]. In the matched analysis, all mismatched pairs favored TESA (b = 17, c = 0); the difference was highly statistically significant (McNemar exact test, two-sided *p* = 1.53 × 10⁻⁵).

Effect size measures are summarized in Table 4. The absolute risk difference was 29.3% (95% Newcombe/Wilson CI: 16.0%–42.9%), and the number needed to treat (NNT) was calculated as 3.4 (95% CI: 2.3–6.3). Since no live births were observed in the ejaculate group, the point estimates for relative risk and odds ratio converge to infinity; however, using the Koopman method, the lower limit of the 95% confidence interval for RR was 2.0, and using the Baptista-Pike method, the lower limit for OR was 6.0.

**Table 4:** Paired comparison of clinical outcomes between EJAC-ICSI and TESA-ICSI cycles (*n* = 58).

Outcome	EJAC-ICSI	TESA-ICSI	*p* value	Effect size (95% CI)
ET	21/58 (36.2%)	30/58 (51.7%)	0.035	-
CP	4/58 (6.9%)	18/58 (31.0%)	**0** **.** **0026**	Conditional OR 5.67 (1.64–30.18)
Miscarriage	4/4 (100%)	1/18 (5.6%)	**0**.**0007**	OR lower limit 6.59
Live Birth	0/58 (0.0%)	17/58 (29.3%)	1.53 × 10⁻⁵	RD 29.3% (16.0%–42.9%); NNT 3.4 (2.3–6.3)
Live Birth/ET	0/21 (0.0%)	17/30 (56.7%)	<0.0001	RD 56.7%; lower limit of the OR 7.18

CI, confidence interval; RD, risk difference; NNT, number needed to treat; OR, odds ratio; ET, embryo transfer; CP, clinical pregnancy. Significant *p* values are indicated as bold.

When considering only cases where embryo transfer was possible, the live birth rate was 17/30 (56.7%) in the TESA-ICSI group and 0/21 (0.0%) in the EJAC-ICSI group; the difference was found to be highly significant by Fisher’s exact test (two-sided *p* < 0.0001; 95% lower limit of OR [Baptista-Pike] = 7.18; absolute risk difference 56.7%).

#### 3.4.4 Number of embryos in cases resulting in live births

When TESA-ICSI cycles resulting in live birth (*n* = 17) were compared with the same patients’ previous unsuccessful EJAC-ICSI cycles, the median (IQR) total number of embryos on day 5 was 6 (2–7) in the EJAC-ICSI group and 5 (4–7.5) in the TESA-ICSI group; no statistically significant difference was observed between the two cycles (Wilcoxon paired, exact two-sided *p* = 0.208; Figure 6).

**Figure 6:**
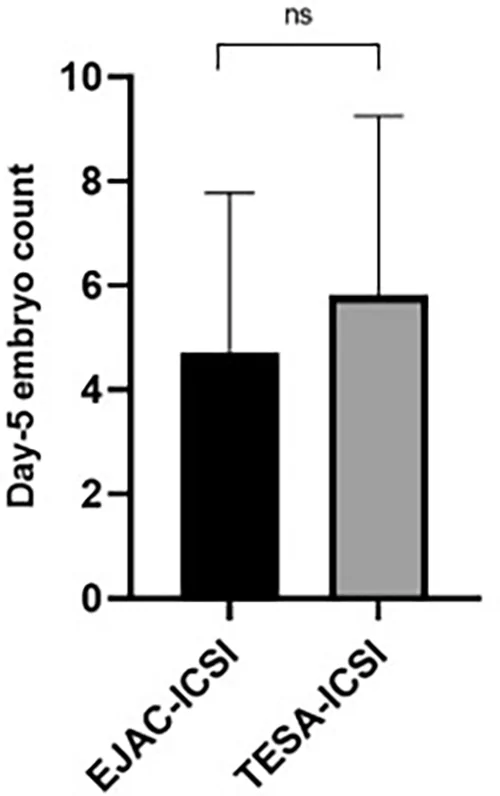
Comparison of the total number of D5 embryos between EJAC-ICSI and TESA-ICSI cycles in the subgroup with live births (*n* = 17) (mean ± SD). The Wilcoxon signed-rank test for matched pairs found no statistically significant difference (exact two-sided *p* = 0.208; ns).

The total number of embryos on Day 6 was observed to be a median of 0.0 (0.0–2.0) in both groups in this subgroup; no significant difference was detected between the two cycles (*p* = 0.574; Figure 7).

**Figure 7:**
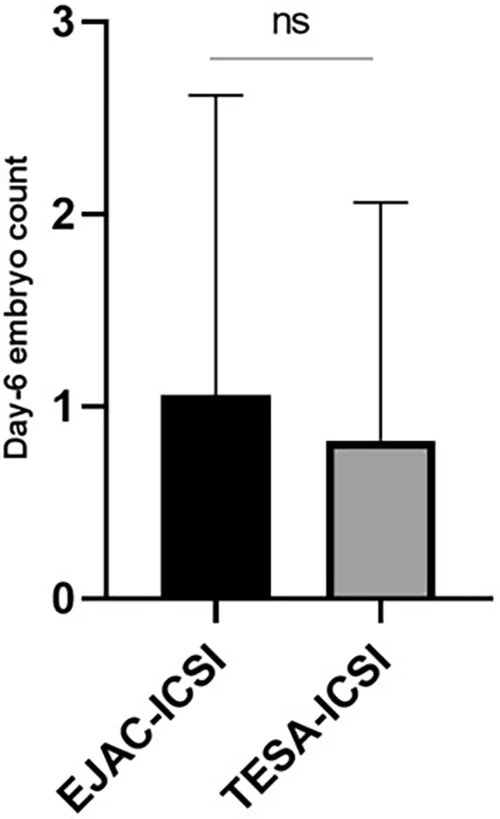
Comparison of the total number of D6 embryos between EJAC-ICSI and TESA-ICSI cycles in the subgroup with live births (*n* = 17) (mean ± SD). The Wilcoxon signed-rank test for matched pairs found no statistically significant difference (exact two-sided *p* = 0.574; ns).

This finding supports the notion that the live birth advantage in the TESA-ICSI cycle stems not from an increase in the number of blastocysts produced, but rather from an improvement in embryo quality (Grade A) and chromosomal integrity (euploidy), and implantation potential.

### 3.5 Sensitivity and subgroup analyses

To evaluate the robustness of the observed live birth advantage of TESA-ICSI, two additional sensitivity analyses were performed (Table 5).

**Table 5:** Sensitivity and subgroup analyses.

Analysis	n	EJAC-ICSI	TESA-ICSI	McNemar exact p
≤6-month interval subgroup				
Clinical pregnancy	35	2 (5.7%)	13 (37.1%)	**0** **.** **003**
Live birth	35	0 (0%)	12 (34.3%)	**<0**.**001**
≤12-month interval subgroup				
Clinical pregnancy	50	4 (8.0%)	16 (32.0%)	**0**.**008**
Live birth	50	0 (0%)	15 (30.0%)	**<0**.**001**

McNemar exact test used for paired binary outcomes. Significant *p* values are indicated as bold.

#### 3.5.1 PGT-A status and subgroup feasibility

PGT-A was performed in at least one cycle in 50 of the 58 couples, and in neither cycle in 8. In these 8 couples no live birth and no clinical pregnancy occurred in either the EJAC-ICSI or the TESA-ICSI cycle, so this subgroup provides no information on whether the live birth benefit persists in the absence of chromosomal selection. Because PGT-A could only be performed when a blastocyst suitable for trophectoderm biopsy was available, PGT-A status is itself downstream of embryo development; subgroups defined by PGT-A status therefore condition on a post-treatment variable and were not analyzed further.

#### 3.5.2 Sensitivity analysis by inter-cycle interval

The median interval between the paired EJAC-ICSI and TESA-ICSI cycles within each couple was 6 months (IQR 3–9; range 2–24, Table 1), with 86% of paired cycles performed within 12 months. To address the potential contribution of secular time drift in laboratory conditions, additional analyses were performed within narrow time windows. In the subgroup of 35 couples whose paired cycles were separated by ≤6 months, the live birth advantage of TESA-ICSI was preserved (12/35 [34.3%] vs. 0/35 [0%]; McNemar exact *p* < 0.001), as was the clinical pregnancy advantage (13/35 [37.1%] vs. 2/35 [5.7%]; McNemar exact *p* = 0.003). The same pattern was observed in the ≤12-month subgroup of 50 couples (live birth: 15/50 [30.0%] vs. 0/50 [0%], McNemar exact *p* < 0.001; clinical pregnancy: 16/50 [32.0%] vs. 4/50 [8.0%], McNemar exact *p* = 0.008, Table 5).

## 4 Discussion

In this within-pair retrospective analysis of 58 sequential ICSI cycle pairs in which an initial EJAC-ICSI cycle had not resulted in a live birth, switching to TESA-ICSI in the subsequent cycle was associated with a marked improvement in reproductive outcomes. Importantly, this benefit was associated with improvements in embryo quality and chromosomal integrity rather than by changes in the number of oocytes retrieved, mature oocytes, or normally fertilized zygotes, which were broadly similar between paired cycles. In contrast, the blastulation rate per 2PN, the proportion of Grade A blastocysts, the rate of euploid embryos on PGT-A, the clinical pregnancy rate, and the live birth rate were all significantly higher with TESA-ICSI, while the miscarriage rate per clinical pregnancy was substantially lower. The advantage in blastocyst morphology was confined to this top category; the proportion of transferable-quality (Grade A or B) blastocysts was similar between cycle types. These observations may support the view that the benefit of TESA-ICSI in this clinical setting is associated with the quality, not the quantity, of the available embryos. The 0% live birth rate in the EJAC-ICSI arm reflects the inclusion criteria, which required that the initial cycle had not resulted in a live birth. Notably, the improvement in clinical outcomes was not accompanied by more embryos being transferred, younger patients, or morphologically better embryos; the proportion of Grade A blastocysts among transferred embryos was in fact higher in the EJAC-ICSI cycles. A euploid embryo was, however, transferred more often in TESA-ICSI cycles, and the contribution of chromosomal selection to the observed outcome difference cannot be separated from that of sperm source in this design.

In the present cohort, the decision to switch to TESA-ICSI was made on clinical and embryological grounds, most often an aneuploid PGT-A result or persistent poor embryo development in the preceding EJAC-ICSI cycle. This empirical approach follows earlier reports in which testicular sperm was used after repeated implantation failure with poor embryo quality, or after cycles with a high proportion of fragmented embryos, without SDF testing (15, 16).

SDF is nonetheless the most frequently invoked biological explanation for outcome differences between the two sperm sources. SDF arises through both testicular mechanisms and post-testicular mechanisms, such as abortive apoptosis, and mostly oxidative stress accumulated during epididymal transit (3). Testicular spermatozoa, retrieved prior to this transit, have been reported to exhibit lower SDF than their ejaculated counterparts; the most recent paired meta-analysis reported a mean difference of approximately 25% normalization in the result (11). The downstream effects of elevated SDF are particularly seen after fertilization: in a large retrospective series of 870 ICSI cycles, higher SDF was associated with lower odds of obtaining a top-quality day-5 blastocyst (7) and similar associations have been documented for blastocyst formation, embryo quality, and miscarriage in additional cohorts (6, 17). The pattern observed in our cohort (unchanged oocyte and total embryo counts, and a lower fertilization rate, alongside improved blastocyst quality, euploidy and live birth) is compatible with such a mechanism. The PGT-A data provide additional insight into the observed differences.

Among cycles tested, the proportion with at least one aneuploid embryo was reduced from approximately 72% with EJAC-ICSI to 35% with TESA-ICSI. This magnitude of effect should be interpreted cautiously given the modest patient numbers, but it is consistent with the hypothesis that a reduction in post-testicular DNA damage may translate into fewer chromosomal abnormalities at the blastocyst stage. The literature on this specific point, however, is not uniform, and three contradictory reports warrant direct discussion. The first is a large cohort study of more than 5,847 ICSI cycles. The research team found that lower sperm concentration and testicular sperm use were significantly associated with lower euploidy per biopsied blastocyst (18). The second is a Turkish cohort of 326 cycles in men with severe male infertility (19),which reported reduced euploidy rates with testicular sperm compared with normozoospermic controls. A key methodological difference distinguishes those reports from the present analysis: both compared testicular sperm cycles, often from men with non-obstructive azoospermia or severe oligozoospermia, with cycles from unselected or normozoospermic controls. Our analysis is, instead, a within-pair comparison restricted to non-azoospermic, non-oligospermic, and non-cryptozoospermic men with a documented prior EJAC-ICSI failure. The third report is methodologically closer to the present study. In 27 couples who underwent an EJAC-ICSI cycle that did not result in a live birth followed by a testicular-sperm cycle, with PGT-A performed in both cycles, blastocyst quality improved significantly with testicular sperm whereas the aneuploidy rate was unchanged (20). Our blastocyst-quality findings are therefore concordant with that study, while our euploidy findings are not. Two differences may be relevant: aneuploidy was expressed per inseminated oocyte and per biopsied embryo in that cohort, whereas the present analysis was conducted at the patient level; and our cohort excluded men with oligozoospermia or cryptozoospermia. The chromosomal advantage observed here should therefore be regarded as preliminary and requires confirmation in larger cohorts using embryo-level denominators. Conversely, in populations with severe spermatogenic failure (such as severe azoospermia), the testicular spermatozoa often carry an intrinsically high rate of aneuploidy due to impaired spermatogenesis, which can negate or even reverse this benefit (21).

The improvements observed in clinical pregnancy, live birth, and miscarriage rates are consistent with the two most recent systematic reviews and meta-analyses, which reported live birth odds ratios of 2.40 (*n* = 446 embryo transfers) (11) and 3.10 (12), and with the work of Greco and colleagues (9) and the seminal meta-analysis (10). However, the evidence base is not uniform. A randomized trial involving oligozoospermic men showed no statistically significant advantage in live birth rates with testicular sperm compared with ejaculated sperm (8). The discrepancy is most plausibly explained by differences in patient selection, sperm retrieval technique (TESA vs. m-TESE), and methodological study design (20, 22).

The principal strength of this study lies in its paired design: each cycle pair was performed in the same couple, eliminating variation in ovarian reserve, endometrial receptivity, transfer protocol, and other female and clinical factors that complicate between-subject comparisons. It is also uncommon in the published literature to find a single dataset that integrates embryo development, PGT-A results, and live birth. Several limitations must, however, be acknowledged.

The study is retrospective and single-center, and the cohort size, while internally consistent, limits subgroup analyses; the analysis of live birth per embryo transfer, in particular, relied on small denominators. PGT-A was not performed in every cycle, so the euploidy comparison does not extend to the entire cohort. Moreover, because PGT-A required a blastocyst suitable for trophectoderm biopsy, PGT-A status is partly determined by embryo development itself; the contribution of chromosomal selection could therefore not be separated from that of sperm source in this dataset. Because no live births occurred in the EJAC-ICSI arm by inclusion criteria, relative risk and odds ratio for this endpoint are not estimable; the absolute risk difference and number needed to treat are the more appropriate effect measures. Furthermore, SDF was not measured in either cycle; these findings are consistent with the hypothesis that testicular spermatozoa may possess superior genomic integrity, but direct evidence was not available in this cohort, and the observed benefit could equally reflect other properties of testicular spermatozoa, such as chromatin packaging or epigenetic signatures.

A separate limitation concerns the sequential design. In this design, the TESA-ICSI cycle followed the EJAC-ICSI cycle. The ideal comparator would have been a concurrent group of couples proceeding to a further EJAC-ICSI cycle rather than to TESA-ICSI. It should be noted, however, that all couples in this cohort had already undergone at least one unsuccessful IVF/ICSI attempt elsewhere before the EJAC-ICSI cycle analyzed here; the EJAC-ICSI cycle therefore itself represents a repeat attempt with ejaculated sperm, and no live birth was obtained in any of these cycles. Part of the observed improvement may nevertheless reflect unmeasured factors related to the subsequent attempt itself, and this possibility cannot be excluded from the present data. Sensitivity analyses restricted to pairs with shorter inter-cycle intervals were reassuring but cannot substitute for a concurrent control group, and prospective studies including a parallel EJAC-ICSI arm are required.

In couples with a prior EJAC-ICSI cycle that did not result in a live birth, a subsequent TESA-ICSI cycle in the same couple was associated with significantly higher blastocyst quality, euploidy, clinical pregnancy, and live birth rates, and with significantly lower miscarriage rates, in the absence of any change in oocyte or fertilization numbers. These findings suggest that TESA-ICSI may be considered as a potential alternative for couples experiencing prior EJAC-ICSI failure, providing a valuable option for individualized patient management.

## Data Availability

The raw data supporting the conclusions of this article will be made available by the authors, without undue reservation.
